# Identification of Residues Important for the Activity of *Haloferax volcanii* AglD, a Component of the Archaeal N-Glycosylation Pathway

**DOI:** 10.1155/2010/315108

**Published:** 2010-05-06

**Authors:** Lina Kaminski, Jerry Eichler

**Affiliations:** Department of Life Sciences, Ben Gurion University, P. O. Box 653, Beersheva 84105, Israel

## Abstract

In *Haloferax volcanii*, AglD adds the final hexose to the N-linked pentasaccharide decorating the S-layer glycoprotein. Not knowing the natural substrate of the glycosyltransferase, together with the challenge of designing assays compatible with hypersalinity, has frustrated efforts at biochemical characterization of AglD activity. To circumvent these obstacles, an in vivo assay designed to identify amino acid residues important for AglD activity is described. In the assay, restoration of AglD function in an *Hfx. volcanii aglD* deletion strain transformed to express plasmid-encoded versions of AglD, generated through site-directed mutagenesis at positions encoding residues conserved in archaeal homologues of AglD, is reflected in the behavior of a readily detectable reporter of N-glycosylation. As such Asp110 and Asp112 were designated as elements of the DXD motif of AglD, a motif that interacts with metal cations associated with nucleotide-activated sugar donors, while Asp201 was predicted to be the catalytic base of the enzyme.

## 1. Introduction

Although the presence of N-glycosylated proteins in Archaea has been known for over 30 years [1], the pathways responsible for this posttranslational modification have only recently been addressed. In *Methanococcus voltae*, *Methanococcus maripaludis,* and *Haloferax volcanii*, products of the *agl* genes have been shown to participate in the assembly of oligosaccharides decorating various glycoproteins in these species [2–4]. At present, however, apart from the oligosaccharyltransferase, AglB [5–7], virtually nothing is known of the catalytic workings of the different Agl proteins. Of the *Hfx. volcanii* Agl proteins identified to date, at least five (i.e., AglD, AglE, AglG, AglI, and AglJ) are predicted to act as glycosyltransferases (GTs), enzymes that catalyze the formation of glycosidic bonds through the transfer of the sugar moieties from nucleotide-activated saccharides to appropriate targets [8]. 

 Based on their amino acid similarities, GTs can be classified into 91 family groups (http://www.cazy.org/fam/acc_GT.html; January, 2009), varying in size and number of functions fulfilled by family members [9, 10]. Furthermore, the different GT families can be clustered based on whether the canonical GT-A or GT-B fold is employed and whether sugar stereochemistry is retained or inverted upon addition of a glycosyl donor [11]. Still, the ability to predict the function of a given GT or to define its catalytic mechanism remains a challenge. This is particularly true in the case of the GT2 family, an ancient group of GT-A fold-bearing GTs containing over 10,000 members derived from various sources and serving at least 12 distinct functions [11, 12]. Like all GT-A fold-bearing GTs, GT2 family members contain a DXD signature motif, shown to interact with a divalent cation (usually Mg^2+^ or Mn^2+^) that facilitates the leaving of the nucleoside diphosphate group of a nucleotide-activated sugar donor as part of the S_N_2-like displacement mechanism believed to be employed by these enzymes [11, 13–17]. The DXD motif also serves to divide the GT-A fold into two portions. The N-terminal portion, containing the sequence that assigns the protein to the GT2 family [18], binds the nucleotide-activated sugar donor [19–22]. By contrast, the C-terminal portion is highly variable and generally serves to recognize the acceptor [17]. Despite such variability, the C-terminal portion of GT2 family members includes a conserved Asp or Glu residue that presumably serves as the catalytic base, thought to assist in the protonation of the nucleophilic hydroxyl group of the acceptor saccharide [11, 21, 23–25]. 

 The GT2 glycosyltransferase family includes *Hfx. volcanii* AglD, previously shown to participate in adding the final hexose to the pentasaccharide comprising two hexoses, two hexuronic acids, and a methylated ester of hexuronic acid decorating at least two sequons of the S-layer glycoprotein [26, 27]. However, due to the fact that its natural substrates have yet to be defined and the challenge of devising in vitro assays for haloarchaeal enzymes due to their hypersaline requirements, little is known of the catalytic workings of AglD. Towards remedying the situation, an in vivo approach has been developed in which the ability of plasmid-encoded versions of AglD, modified through site-directed mutagenesis, to restore the absent function to an *aglD* deletion strain, was tested. Results obtained employing this novel assay point to Asp110-Thr111-Asp112 as corresponding to the DXD motif and Asp201 as corresponding to the catalytic base of* Hfx. volcanii* AglD.

## 2. Methods

### 2.1. Strains and Growth Conditions

The *Hfx. volcanii* background strain WR536 (H53) and the same strain deleted for* aglD* were grown in complete medium containing 3.4 M NaCl, 0.15 M MgSO_4_·7H_2_0, 1 mM MnCl_2_, 4 mM KCl, 3 mM CaCl_2_, 0.3% (w/v) yeast extract, 0.5% (w/v) tryptone and 50 mM Tris-HCl, pH 7.2, at 42°C [28]. A complete description of the *aglD* deletion strain and the protocol used to delete the gene have been previously published [5].

### 2.2. In Vivo AglD Assay

To assay AglD activity, *Hfx. volcanii* cells deleted of *aglD* [5] were transformed to express plasmid-encoded versions of AglD that included an N-terminally fused *Clostridium thermocellum* cellulose-binding domain (CBD) [29]. To introduce nonnative residues into AglD, the plasmid-encoded version of *aglD* (GenBank accession number CAM91696.1) was modified by site-directed mutagenesis. Restoration of AglD function lost as a result of deletion of the genomic copy of the encoding gene was determined by the ability of the transformed cells to reverse the enhanced SDS-PAGE migration of the S-layer glycoprotein and loss of PAS glycostaining of the same reporter, that is, novel traits of the S-layer glycoprotein that appeared in cells lacking AglD.

### 2.3. Site-Directed Mutagenesis

Mutated versions of *aglD* were generated by site-directed mutagenesis using the Quikchange (Stratagene) protocol, performed according to the manufacturer's instructions, with plasmid pWL-CBD-AglD, encoding CBD-AglD [29], serving as template. Oligonucleotide primers used to introduce the various mutations are listed in Supplementary Table  1 (available online at doi:10.1155/2010/315108). The introduction of mutations was confirmed by sequencing, performed both before and following introduction of plasmid-encoded mutated *aglD* into *Hfx. volcanii*.

### 2.4. Other Methods

Periodic acid-Schiff (PAS) reagent glycoprotein staining was performed as described previously [30]. Immunoblots were performed using polyclonal antibodies raised against the *C. thermocellum* CBD (obtained from Ed Bayer, Weizmann Institute of Science; 1 : 10,000). Antibody binding was detected using goat antirabbit horseradish peroxidase-(HRP-) conjugated antibodies (1 : 4000, BioRad, Hercules, CA) and an ECL-enhanced chemiluminescence kit (Amersham, Buckingham, UK).

## 3. Results

### 3.1. AglD Activity in AglD-Deleted *Hfx. volcanii* Cells Is Restored Upon Complementation with Plasmid-Encoded AglD

As a first step towards describing AglD function, efforts were directed at creating an assay to allow for characterization of the activity of the enzyme. Accordingly, cells deleted of the encoding gene were transformed with a plasmid encoding a version of the protein designed to include an N-terminally fused *Clostridium thermocellum* cellulose-binding domain (CBD) [29]. As previously reported [29], an 85 kDa band, corresponding to the predicted molecular mass of the 17 kDa CBD moiety and the 68 kDa AglD protein, was expressed in the transformed cells and recognized in an immunoblot using antiCBD antibodies (not shown). 

**Figure 1:**
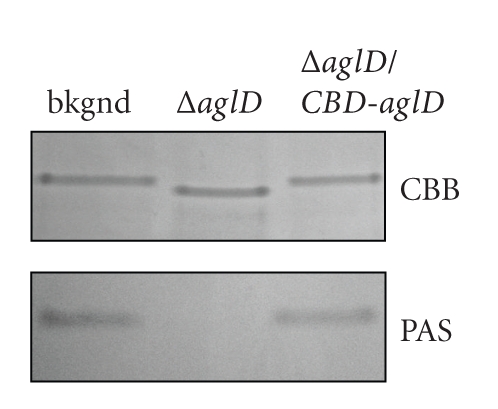
*aglD*-complemented *Hfx. volcanii* cells regain the ability to properly glycosylate the S-layer glycoprotein. The protein contents of cells of the WR536 background strain (bkgnd), the same strain deleted of *aglD *(Δ*aglD*) or the AglD-lacking strain transformed with a plasmid encoding CBD-AglD (Δ*aglD/CBD-aglD*) were separated by 5% SDS-PAGE and the S-layer glycoprotein was detected by Coomassie stain (CBB) or periodic acid-Schiff (PAS) reagent. In the presence of CBD-AglD, the migration and positive glycostaining of the S-layer glycoprotein are as observed in the background strain.

 Deletion of *aglD* results in the absence of the final hexose of the pentasaccharide decorating the *Hfx. volcanii* S-layer glycoprotein [26]. As such, the S-layer glycoprotein in the deletion strain migrates faster in SDS-PAGE than does the native protein in the background strain [5]. Moreover, the S-layer glycoprotein is not recognized by PAS glycostain in the mutant strain. However, as reflected in Figure 1, the S-layer glycoprotein from cells of the *aglD*-deleted strain transformed to express CBD-tagged AglD migrated to the same position as did the protein from the background strain and was similarly PAS-stained. As such, complementation of *Hfx. volcanii* cells lacking *aglD* with an AglD-encoding plasmid restores the absent activity to the deletion strain. 

 To demonstrate the involvement of a given AglD residue in the activity of the enzyme, the return of AglD activity to the *aglD* deletion strain, upon introduction of a plasmid-encoded version of AglD modified at the amino acid position in question, was assessed. In these experiments, the SDS-PAGE migration of the S-layer glycoprotein from cells of the background strain, from cells deleted of *aglD*, and from AglD-lacking cells transformed to express select mutant AglD proteins was addressed. In addition, the S-layer glycoprotein in each of the three populations of *Hfx. volcanii* cells was subjected to PAS glycostaining. While this in vivo approach cannot distinguish between residues necessary for catalytic activity from those important for proper AglD folding, it, nonetheless, offers a facile route for identifying important AglD residues until such time as AglD activity can be directly assayed in vitro.

### 3.2. Identification of Conserved AglD Residues

**Figure 2:**
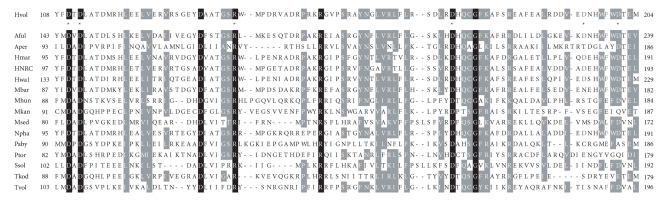
Conserved residues in archaeal AglD proteins. The sequences of *Hfx. Volcanii  *AglD (accession number AM698042) and AglD homologues in *Archaeoglobus fulgidus* (NP_069415.1; Aful), *Aeropyrum pernix* (NP_147774.1; Aper), *Haloarcula marismortui* (YP_136461.1; Hmar), *Halobacterium* sp. NRC-1 (NP_279416.1; HNRC), *Haloquadratum walsbyi *(YP_657261.1; Hwal), *Methanosarcina acetivorans* (NP_618739.1; Mace), *Methanosarcina barkeri *(YP_304067.1; Mbar), *Methanospirillum hungatei* (YP_503949.1; Mhun), *Methanopyrus kandleri* (NP_614163.1; Mkan),* Metallosphaera sedula* (YP_001191894; Msed), *Natronomonas pharaonis* (YP_326773.1; Npha), *Pyrococcus abyssi* (NP_127133.1; Paby), *Picrophilus torridus* (YP_024256.1; Ptor), *Sulfolobus solfataricus* (NP_342803.1; Ssol), *Thermococcus kodakarensis* (YP_182777.1; Tkod), and *Thermoplasma volcanium* (NP_111403.1; Tvol) were aligned by ClustalW2 (www.ebi.ac.uk/Tools/clustalw2/index.html), using the default settings. The region of the highest similarity is shown. Completely conserved residues are shown against a black background, while largely conserved residues (i.e., similar residues conserved in at least 11 sequences) are shown against a grey background. Amino acid numbers are shown at the start and end of each sequence. Asterisks are placed under *Hfx. volcanii* AglD D110, D112, R139, D173, D195, and D201 (see text for details).

To select candidate residues for site-directed mutagenesis, the *Hfx. volcanii* AglD sequence was aligned with selected homologous archaeal sequences using ClustalW (http://www.ebi.ac.uk/Tools/clustalw2/index.html). It should be noted, however, that it is not yet known whether the various homologues considered indeed catalyze the same reaction as does *Hfx. volcanii* AglD. Indeed, it remains to be confirmed that N-glycosylation occurs in all of the species listed. Nonetheless, such alignment revealed the presence of a stretch of amino acids in the N-terminal region of AglD showing substantial overlap with similarly situated regions in the various archaeal homologues considered (Figure 2). In the *Hfx. volcanii* protein, this stretch corresponds to the region between Asp110 and Glu203, a portion of the protein previously localized to the cytoplasm [29] and which includes seven residues absolutely conserved in the sequences considered, namely, Asp110, Asp112, Asp133, Arg139, Arg152, Asp173, and Gly177. Between Asp133 and Arg139, between Trp198 and Glu203, and in the region surrounding Asp173 and Gly177, several highly conserved residues were also detected. To determine whether any of these residues contribute to AglD function, the corresponding *aglD* codons were modified by site-directed mutagenesis using the primer pairs listed in Supplementary Table 1, and the ability of plasmid-encoded versions of the mutant proteins to restore AglD function in the *aglD* deletion strain was considered.

### 3.3. The DXD Catalytic Motif of AglD Likely Comprises Asp110 and Asp 112

**Figure 3:**
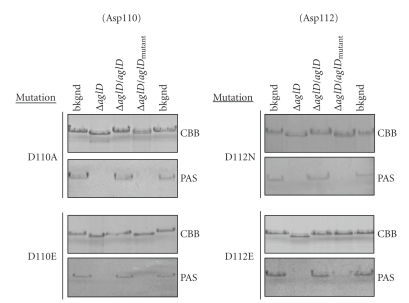
*Hfx. volcanii* AglD Asp110 and Asp112 residues likely participate in the GT2 DXD motif involved in the catalytic activity of the enzyme. Site-directed mutagenesis was performed to generate CBD-AglD containing mutations of Asp110 (left column) or Asp112 (right column), as listed on the left of each panel. For each mutant, the upper and lower panels, respectively, show the Coomassie- and PAS-stained S-layer glycoprotein from the background strain (lanes 1 and 5), from the *aglD* deletion strain (lane 2), from the *aglD* deletion strain complemented with a plasmid encoding CBD-AglD (lane 3), or from the *aglD* deletion strain complemented with a plasmid encoding CBD fused to mutated AglD (lane 4).

The GT-A fold found in GT2 family members includes a DXD motif that contributes to the catalytic activity of the enzyme [11, 13, 14, 16, 17]. Sequence alignment-based examination of the *Hfx. volcanii *AglD sequence points to Asp110-Thr111-Asp112 as comprising this motif (Figure 2). To directly test this hypothesis, the site-directed mutagenesis approach described above was enlisted. Figure 3 addresses the effects of replacing either AglD Asp110 or Asp112 with other residues. Transformation of AglD-lacking cells to express AglD D110A resulted in both the failure of plasmid-encoded AglD to restore S-layer glycoprotein migration to the position of this reporter in the background strain as well as the lost ability of PAS glycostain to label the S-layer glycoprotein. The same was true in cells transformed to express AglD D110E, a mutation that retains the negative charge at this position (Figure 3), or upon introduction of an Asn residue at this position (not shown). 

 When AglD Asp112 of the plasmid-encoded protein was replaced with an Asn, no recovery of AglD function in the transformed *aglD* deletion strain was realized, reflected in the inability of cells expressing the mutated version of AglD to restore SDS-PAGE migration and PAS glycostaining of the S-layer glycoprotein, as realized in the background strain (Figure 3). By contrast, transformation of the deletion strain to express AglD D112E led to a restoration of SDS-PAGE migration of the S-layer glycoprotein to the position seen in background cells but only a partial recovery (6% ± 0.5% (standard deviation), *n* = 3) of PAS glycostaining (Figure 3). 

 The importance of Asp110 and Asp112 for *Hfx. volcanii* AglD activity points to these two residues as comprising the DXD motif found in GT-A fold-bearing GTs. However, while the Asp residue at position 110 is apparently essential for activity, the presence of Glu at position 112 yields a functional enzyme that apparently acts differently from the native enzyme, as reflected in the limited PAS staining detected with this mutant.

### 3.4. Asp201 Is Likely the Catalytic Base of AglD

In addition to the DXD motif considered above, the activity of GT2 family members also relies on an Asp or Glu residue found in the acceptor-binding domain of the protein. First identified in the solved three-dimensional structure of *Bacillus subtilis* SpsA as Asp191 [20], this residue and its equivalents in other GTs are thought to serve as the base catalyst in the direct displacement mechanism apparently employed by these enzymes [11, 20]. To identify the *Hfx. volcanii* AglD equivalent of *B. subtilis* SpsA Asp191, the sequence of the soluble region of AglD (residues 1–259) was aligned with the sequence of *B. subtilis* SpsA, as well as with those of the other GT2 enzymes where the functional equivalent of *B. subtilis* SpsA Asp191 is known, namely, *Sinorhizobium meliloti* ExoM and *Salmonella enterica* WbbE. Earlier site-directed mutagenesis efforts had revealed ExoM Asp187 and WbbE Glu180 to serve the same role as SpsA Asp191 [21, 22]. Tcoffee (http://tcoffee.vital-it.ch/cgi-bin/Tcoffee/tcoffee_cgi/index.cgi?stage1=1anddaction=TCOFFEE::Regular) aligned AglD Asp201 with SpsA Asp191, ExoM Asp187 and WbbE Glu180. The MAFFT program (v6.531b; http://www.ebi.ac.uk/Tools/mafft/index.html) also aligned AglD Asp201 with the same SpsA, ExoM and WbbE residues. On the other hand, alignment of archaeal homologues of *Hfx. volcanii* AglD using the ClustalW (Figure 2), Tcoffee or MAFFT programs revealed that AglD Asp173 but not Asp201 is conserved. In all cases, the programs consulted were used with their default settings. 

 As a next step towards identifying the AglD equivalent of SpsA Asp191, the importance of Asp173, Asp195, and Asp201 for AglD activity was considered by site-directed mutagenesis. 

**Figure 4:**
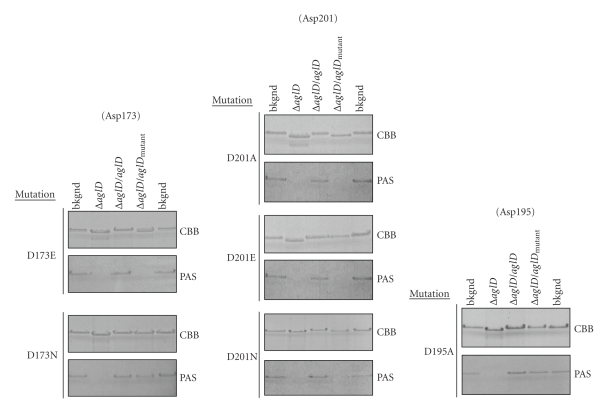
*Hfx. volcanii* AglD Asp201 is apparently the catalytic base of the enzyme. Site-directed mutagenesis was performed to generate CBD-AglD containing mutations of Asp173 (upper left column), Asp195 (lower left column), and Asp201 (right column), as listed on the left of each panel. For each mutant, the upper and lower panels, respectively, show the Coomassie- and PAS-stained S-layer glycoprotein from the background strain (lanes 1 and 5), from the *aglD* deletion strain (lane 2), from the *aglD* deletion strain complemented with a plasmid encoding CBD-AglD (lane 3), or from the *aglD* deletion strain complemented with a plasmid encoding CBD fused to mutated AglD (lane 4).

 Transformation of *Hfx. volcanii *Δ*aglD* cells with a plasmid encoding AglD D173E (Figure 4) did not restore the SDS-PAGE migration or glycostaining of the S-layer glycoprotein. However, as these S-layer glycoprotein traits were fully restored in cells expressing AglD D173N, it would appear that the conserved Asp173 does not serve as the catalytic base of the enzyme but is likely important for AglD structure. This idea may be supported by the observation that the D173A mutant could not be expressed (not shown). The importance of Asp195, another somewhat conserved Asp residue in this region, was also considered. *Hfx. volcanii *Δ*aglD* cells transformed to express AglD D195A (Figure 4) or D195E (not shown) readily replaced the actions of the missing enzyme, showing that the Asp at this position is not necessary for AglD activity. By contrast, if the *aglD*-deleted strain was transformed to express AglD D201A or D201N, SDS-PAGE migration and glycostaining of the S-layer glycoprotein were as observed in cells lacking native AglD (Figure 4). When, however, *Hfx. volcanii *Δ*aglD* cells were transformed to express the D201E mutant, the S-layer glycoprotein behaved as in the background strain (Figure 4). These results thus point to Asp201 as being the catalytic base of AglD and the functional equivalent of SpsA Asp191. Furthermore, as is the case with other GT2 family members [22], Asp201 can be replaced by Glu. 

**Figure 5:**
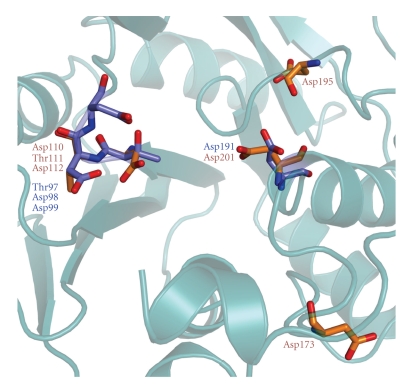
Homology modeling of *Hfx. volcanii* AglD residues based on the available three-dimensional structure of *B. subtilis* SpsA. Structural modeling was performed by using the SWISS-MODEL program (http://swissmodel.expasy.org/) and visualized using PyMol (http://www.pymol.org/). *B. subtilis* SpsA Thr97, Asp98, Asp99, and Asp191 are shown in blue, while *Hfx. volcanii* AglD Asp110, Thr111, Asp112, Asp173, Asp195, and Asp 201 are shown in brown. The ribbon diagram in the background corresponds to the three-dimensional structure of SpsA [20]. The RMS value, reflecting the quality of the homology modeling, is 0.61 angstroms.

 Homology modeling of *Hfx. volcanii* AglD residues Asp110-Asp112, Asp173, Asp195, and Asp201, based on the available three-dimensional structural of *B. subtilis* SpsA [20], further supports the assignment of AglD D201 as being equivalent to subtilis SpsA Asp191. As reflected in Figure 5, considerable overlap in term of both position and orientation exists between AglD D201 and *B. subtilis* SpsA Asp191. The same cannot be said for either AglD Asp173 or Asp195.

### 3.5. The Conserved Arg139 Residue Is Needed for AglD Activity

**Figure 6:**
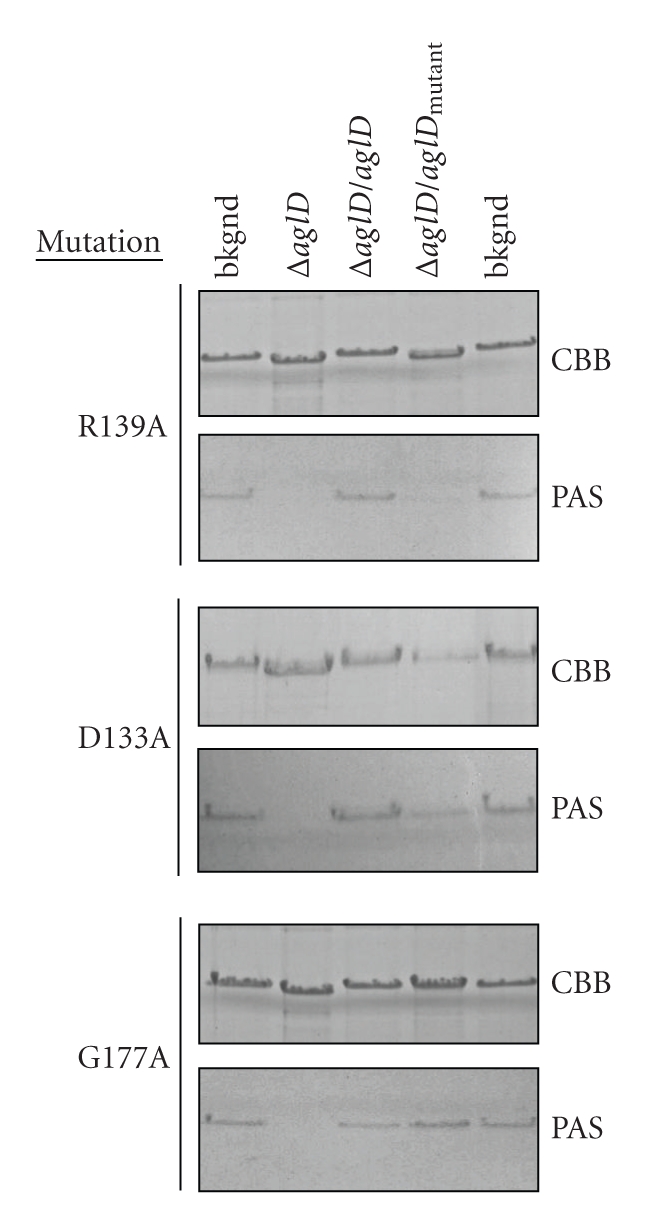
The conserved Arg139 residue is needed for AglD activity, unlike the conserved Asp133 or Gly177 residues. Site-directed mutagenesis was performed to generate CBD-AglD containing mutations of Asp133, Arg139, and Gly177, as listed on the left of each panel. For each mutant, the upper and lower panels respectively show the Coomassie- and PAS-stained S-layer glycoprotein from the background strain (lanes 1 and 5), from the *aglD* deletion strain (lane 2), from the *aglD* deletion strain complemented with a plasmid encoding CBD-AglD (lane 3), or from the *aglD* deletion strain complemented with a plasmid encoding CBD fused to mutated AglD (lane 4).

*Hfx. volcanii* AglD and its archaeal homologues also contain several other fully conserved residues in that part of the soluble N-terminal region under consideration in this study. The contribution of these residues, as well as that of their neighbors, was next considered. Complementation of Δ*aglD Hfx. volcanii* cells with plasmid-encoded AglD R139A failed to restore either S-layer glycoprotein migration in SDS-PAGE or the ability of PAS glycostain to label this reporter (Figure 6). The same was true in cells expressing AglD R139E, R139K or R139M (not shown). Thus, AglD Arg139 is apparently essential for enzyme activity. By contrast, complementation of AglD-lacking *Hfx. volcanii* cells with plasmid-encoded AglD D133A or G177A restored S-layer glycoprotein SDS-PAGE migration to that observed for the native protein, although less S-layer glycoprotein is detected in the cells expressing AglD D133A. The significance of this observation is not clear. In addition, the S-layer glycoprotein in both AglD D133A- and G177A-expressing cells could be glycostained (Figure 6). As such, although conserved in *Hfx. volcanii* AglD and its archaeal homologues, Asp133 and Gly177 do not appear to be essential for the catalytic workings of the enzyme. Similarly, the introduction of CBD-tagged AglD G137A, S138A, Q175A, C176A, F178A or K179A mutants into *Hfx. volcanii *Δ*aglD* cells led to a restoration of AglD activity, indicating that none of these residues contribute to the reaction catalyzed by the enzyme (not shown). 

**Figure 7:**
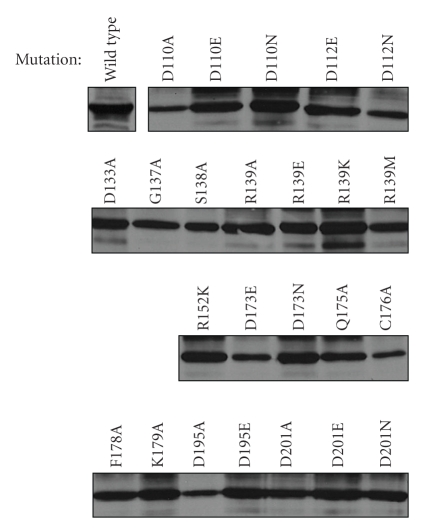
Expression levels of the various versions of CBD-AglD. *Hfx. volcanii* cells expressing the various AglD mutants considered in this study were grown to OD_550_ 1.0 and their protein contents were separated on 10% SDS-PAGE. The CBD-AglD content of each strain was subsequently assessed by immunoblot using polyclonal antiCBD antibodies. Antibody binding was detected using HRP-conjugated secondary antibodies and an enhanced chemiluminescence kit.

 Finally, to eliminate the possibility that the inability of certain plasmid-encoded versions of the protein to restore absent AglD activity was due to poor or no expression, the level of each CBD-AglD considered in this study was assessed by immunoblot using antiCBD antibodies (Figure 7).

## 4. Discussion

When one considers that *Nanoarchaeum equitans*, the archaeon containing the smallest genome identified to date [31, 32], encodes only 3 GTs, namely, one member of the GT2 family and two members of the GT4 family [33], it is fair to say that analysis of archaeal GTs can provide unique insight into the evolution of such enzymes, as well as adding to our comprehension of protein processing in extreme conditions. Despite such promise, only limited experimental data on archaeal GTs involved in protein glycosylation is presently available. The crystal structure of Stt3/AglB from *Pyrococcus furiosus*, the sole component of the archaeal oligosaccharyltransferase [5, 6], has been solved [7], shedding new light on the workings of this central component of the N-glycosylation machinery. Still, although* P. furiosus* has been reported to contain glycoproteins [34], the oligosaccharyltransferase in this species has been thus far only demonstrated to modify an artificial substrate [7, 35]. Similarly, while both *Thermoplasma acidophilum* [36] and *Pyrococcus horikoshii* [37] have been reported to contain glycoproteins, the participation of biochemically characterized dolichyl phosphomannose synthases from these species [37, 38] in protein glycosylation has yet to be demonstrated. As such, the present analysis of *Hfx. volcanii* AglD represents the first examination of a glycosyltransferase experimentally verified as participating in the modification of an identified archaeal glycoprotein, namely, the S-layer glycoprotein. 

 In the present study, sequence alignment was combined with site-directed mutagenesis to identify AglD residues important for the function of the enzyme, as reflected in AglD-mediated modulation of the SDS-PAGE migration and glycostaining of a reporter glycoprotein, the S-layer glycoprotein. This approach assigned AglD Asp110, Thr111, and Asp112 as the DXD motif typical of inverting GT-A fold-bearing GTs. AglD Asp110 was shown to be essential for catalytic activity, while a negative charge at position 112 was deemed necessary. In the case of the AglD D112E mutant, recovery of S-layer glycoprotein SDS-PAGE migration was noted, yet the loss of PAS glycostaining associated with the deletion strain was largely not restored. This could reflect the generation of an enzyme possessing different activity than that of the native protein, one that adds a different sugar to the final position of S-layer glycoprotein-bound pentasaccharide. Indeed, the failure of PAS glycostain to label the tetrasaccharide N-linked to the S-layer glycoprotein in cells lacking AglD points to inability of this labeling reagent to interact with certain sugar subunits. Moreover, within the GT2 family (whose members include *Hfx. volcanii* AglD), differences in the organization and importance of DXD motif constituents exist. In the case of *S. meliloti* ExoM, where the DXD motif includes Asp96 and Asp98, it was shown that replacing the former with Ala completely eliminated enzymatic activity, whereas the same replacement at position 98 only led to a 70% loss of activity [21]. In* S. enterica* WbbE, where the DXD motif is expanded to include Asp93, Asp95, and Asp96, it was shown that exchanging either Asp93 or Asp96 with Ala abolished enzyme activity, while the same replacement at Asp95 only reduced that activity [22]. 

 The site-directed mutagenesis approach developed here, along with sequence alignment and homology modeling, also indicate Asp201 as likely serving as the AglD catalytic base. Just as the corresponding residue in *S. enterica* WbbE, that is, Glu180, could be functionally replaced by Asp [22], AglD D201E was also active. By contrast, replacing the Asp187 catalytic base in *S. mililoti* ExoM led to a complete loss of function [21]. Such nuances may be indicative of differences in the donors and/or acceptors employed by each enzyme or point to unique mechanistic traits. In addition, although conserved in the archaeal AglD homologues examined in this study, *Hfx. volcanii* AglD Asp173 was not assigned as the catalytic base of the enzyme, given its functional replacement by a similarly sized Asn but not a similarly charged Glu. Hence, it would appear that Asp173 is of structural, rather than catalytic, importance to AglD activity. In addition to these residues, at least another *Hfx. volcanii* AglD amino acid seems to be needed for enzyme function, that is, Arg139. The AglD counterparts of other residues shown to be important for the catalytic activity of GT2 family members, such as *S. meliloti* ExoM Asp44 and Asp96 [21], may also play a role in the activity of the archaeal enzyme. 

 In conclusion, this paper describes an in vivo assay designed to consider the contribution of various AglD residues to the activity of the enzyme. In the assay, the ability of plasmid-encoded versions of AglD, selectively mutated at positions suspected of being important for enzyme function, to restore both S-layer glycoprotein SDS-PAGE migration and glycostaining affected in Δ*aglD* cells is assessed. In this manner, Asp110, Asp112, and Asp201 were all determined as being important for AglD activity, as was Asp139.

## Supplementary Material

Supplementary Table 1—Primers used for site-directed mutagenesis of aglD.

## References

[1] Mescher MF, Strominger JL (1976). Purification and characterization of a prokaryotic glycoprotein from the cell envelope of *Halobacterium salinarium*. *The Journal of Biological Chemistry*.

[2] Abu-Qarn M, Eichler J, Sharon N (2008). Not just for Eukarya anymore: N-glycosylation in Bacteria and Archaea. *Current Opinion in Structural Biology*.

[3] Yurist-Doutsch S, Chaban B, VanDyke DJ, Jarrell KF, Eichler J (2008). Sweet to the extreme: protein glycosylation in Archaea. *Molecular Microbiology*.

[4] VanDyke DJ, Wu J, Logan SM (2009). Identification of genes involved in the assembly and attachment of a novel flagellin N-linked tetrasaccharide important for motility in the archaeon *Methanococcus maripaludis*. *Molecular Microbiology*.

[5] Abu-Qarn M, Eichler J (2006). Protein N-glycosylation in Archaea: defining *Haloferax volcanii* genes involved in S-layer glycoprotein glycosylation. *Molecular Microbiology*.

[6] Chaban B, Voisin S, Kelly J, Logan SM, Jarrell KF (2006). Identification of genes involved in the biosynthesis and attachment of *Methanococcus voltae* N-linked glycans: insight into N-linked glycosylation pathways in Archaea. *Molecular Microbiology*.

[7] Igura M, Maita N, Kamishikiryo J (2008). Structure-guided identification of a new catalytic motif of oligosaccharyltransferase. *EMBO Journal*.

[8] Taniguchi N, Ekuni A, Ko JH (2001). A glycomic approach to the identification and characterization of glycoprotein function in cells transfected with glycosyltransferase genes. *Proteomics*.

[9] Coutinho PM, Deleury E, Davies GJ, Henrissat B (2003). An evolving hierarchical family classification for glycosyltransferases. *Journal of Molecular Biology*.

[10] Cantarel BI, Coutinho PM, Rancurel C, Bernard T, Lombard V, Henrissat B (2009). The Carbohydrate-Active EnZymes database (CAZy): an expert resource for glycogenomics. *Nucleic Acids Research*.

[11] Lairson LL, Henrissat B, Davies GJ, Withers SG (2008). Glycosyl transferases: structures, functions, and mechanisms. *Annual Review of Biochemistry*.

[12] Henrissat B, Sulzenbacher G, Bourne Y (2008). Glycosyltransferases, glycoside hydrolases: surprise, surprise!. *Current Opinion in Structural Biology*.

[13] Breton C, Bettler E, Joziasse DH, Geremia RA, Imberty A (1998). Sequence-function relationships of prokaryotic and eukaryotic galactosyltransferases. *Journal of Biochemistry*.

[14] Wiggins CA, Munro S (1998). Activity of the yeast MNN1 alpha-1,3-mannosyltransferase requires a motif conserved in many other families of glycosyltransferases. *Proceedings of the National Academy of Sciences of the United States of America*.

[15] Breton C, Imberty A (1999). Structure/function studies of glycosyltransferases. *Current Opinion in Structural Biology*.

[16] Unligil UM, Rini JM (2000). Glycosyltransferase structure and mechanism. *Current Opinion in Structural Biology*.

[17] Breton C, Šnajdrová L, Jeanneau C, Koča J, Imberty A (2006). Structures and mechanisms of glycosyltransferases. *Glycobiology*.

[18] Campbell JA, Davies GJ, Bulone V, Henrissat B (1997). A classification of nucleotide-diphospho-sugar glycosyltransferases based on amino acid sequence similarities. *Biochemical Journal*.

[19] Saxena IM, Brown RM, Fevre M, Geremia RA, Henrissat B (1995). Multidomain architecture of *β*-glycosyl transferases: implications for mechanism of action. *Journal of Bacteriology*.

[20] Charnock SJ, Davies GJ (1999). Structure of the nucleotide-diphospho-sugar transferase, SpsA from Bacillus subtilis, in native and nucleotide-complexed forms. *Biochemistry*.

[21] Garinot-Schneider C, Lellouch AC, Geremia RA (2000). Identification of essential amino acid residues in the *Sinorhizobium meliloti* glucosyltransferase ExoM. *The Journal of Biological Chemistry*.

[22] Keenleyside WJ, Clarke AJ, Whitfield C (2001). Identification of residues involved in catalytic activity of the inverting glycosyl transferase WbbE from Salmonella enterica serovar borreze. *Journal of Bacteriology*.

[23] Murray BW, Takayama S, Schultz J, Wong C-H (1996). Mechanism and specificity of human *α*-1,3-fucosyltransferase V. *Biochemistry*.

[24] Pedersen LC, Darden TA, Negishi M (2002). Crystal structure of *β*1,3-glucuronyltransferase I in complex with active donor substrate UDP-GlcUA. *The Journal of Biological Chemistry*.

[25] Kakuda S, Shiba T, Ishiguro M (2004). Structural basis for acceptor substrate recognition of a human glucuronyltransferase, GlcAT-P, an enzyme critical in the biosynthesis of the carbohydrate epitope HNK-1. *The Journal of Biological Chemistry*.

[26] Abu-Qarn M, Yurist-Doutsch S, Giordano A (2007). *Haloferax volcanii* AglB and AglD are involved in N-glycosylation of the S-layer glycoprotein and proper assembly of the surface layer. *Journal of Molecular Biology*.

[27] Magidovich H, Yurist-Doutsch S, Konrad Z (2010). AglP is a S-adenosyl-L-methionine-dependent methyltransferase that participates in the N-glycosylation pathway of *Haloferax volcanii*. *Molecular Microbiology*.

[28] Mevarech M, Werczberger R (1985). Genetic transfer in *Halobacterium volcanii*. *Journal of Bacteriology*.

[29] Plavner N, Eichler J (2008). Defining the topology of the N-glycosylation pathway in the halophilic archaeon *Haloferax volcanii*. *Journal of Bacteriology*.

[30] Dubray G, Bezard G (1982). A highly sensitive periodic acid-silver stain for 1,2-diol groups of glycoproteins and polysaccharides in polyacrylamide gels. *Analytical Biochemistry*.

[31] Huber H, Hohn MJ, Rachel R, Fuchs T, Wimmer VC, Stetter KO (2002). A new phylum of Archaea represented by a nanosized hyperthermophilic symbiont. *Nature*.

[32] Waters E, Hohn MJ, Ahel I (2003). The genome of *Nanoarchaeum equitans*: insights into early archaeal evolution and derived parasitism. *Proceedings of the National Academy of Sciences of the United States of America*.

[33] Magidovich H, Eichler J (2009). Glycosyltransferases and oligosaccharyltransferases in Archaea: putative components of the N-glycosylation pathway in the third domain of life. *FEMS Microbiology Letters*.

[34] Weinberg MV, Schut GJ, Brehm S, Datta S, Adams MWW (2005). Cold shock of a hyperthermophilic archaeon: *Pyrococcus furiosus* exhibits multiple responses to a suboptimal growth temperature with a key role for membrane-bound glycoproteins. *Journal of Bacteriology*.

[35] Kohda D, Yamada M, Igura M, Kamishikiryo J, Maenaka K (2007). New oligosaccharyltransferase assay method. *Glycobiology*.

[36] Yang LL, Haug A (1979). Purification and partial characterization of a procaryotic glycoprotein from the plasma membrane of *Thermoplasma acidophilum*. *Biochimica et Biophysica Acta*.

[37] Urushibata Y, Ebisu S, Matsui I (2008). A thermostable dolichol phosphoryl mannose synthase responsible for glycoconjugate synthesis of the hyperthermophilic archaeon *Pyrococcus horikoshii*. *Extremophiles*.

[38] Zhu BCR, Laine RA (1996). Dolichyl-phosphomannose synthase from the Archae *Thermoplasma acidophilum*. *Glycobiology*.

