# Supplementary material for: Identification of Residues Important for the Activity of Haloferax volcanii AglD, a Component of the Archaeal N-Glycosylation Pathway
Source: Archaea. 2010 May 6;2010:315108. doi: 10.1155/2010/315108 (PMC2877612; doi:10.1155/2010/315108)
Supplement: Supplementary file 1 — Supplementary Table 1—Primers used for site-directed mutagenesis of aglD. [file 315108.f1.pdf]

Supplementary Table 1 – Primers used for site-directed mutagenesis of *aglD*

Mutant	Primers
D110A	CGACACGCTCGTCTACTTC**GCC**ACGGACCTCGCCACGGAC (Fwd)
	GTCCGTGGCGAGGTCCGT**GGC**GAAGTAGACGAGCGTGTCG (Rev)
D110E	GACACGCTCGTCTACTTC**GAG**ACGGACCTCGCCACGGACATG (Fwd)
	CATGTCCGTGGCGAGGTCCGT**CTC**GAAGTAGACGAGCGTGTC (Rev)
D110K	GACACGCTCGTCTACTTC**AAG**ACGGACCTCGCCACGGACATG (Fwd)
	CATGTCCGTGGCGAGGTCCGT**CTT**GAAGTAGACGAGCGTGTC (Rev)
D110N	GACACGCTCGTCTACTTC**AAC**ACGGACCTCGCCACGGACATG (Fwd)
	CATGTCCGTGGCGAGGTCCGT**GTT**GAAGTAGACGAGCGTGTC (Rev)
D112A	CACGCTCGTCTACTTCGACACG**GCC**CTCGCCACGGACATGCGACACC (Fwd)
	GGTGTCGCATGTCCGTGGCGAG**GGC**CGTGTCGAAGTAGACGAGCGTG (Rev)
D112E	CACGCTCGTCTACTTCGACACG**GAG**CTCGCCACGGACATGCGACACC (Fwd)
	GGTGTCGCATGTCCGTGGCGAG**CTC**CGTGTCGAAGTAGACGAGCGTG (Rev)
D112K	CACGCTCGTCTACTTCGACACG**AAG**CTCGCCACGGACATGCGACACC (Fwd)
	GGTGTCGCATGTCCGTGGCGAG**CTT**CGTGTCGAAGTAGACGAGCGTG (Rev)
D112N	CACGCTCGTCTACTTCGACACG**AAC**CTCGCCACGGACATGCGACACC (Fwd)
	GGTGTCGCATGTCCGTGGCGAG**GTT**CGTGTCGAAGTAGACGAGCGTG (Rev)
D133A	GCGTCCGCTCCGGCGAGTAC**GCC**GCCGCGACCGGGTCGCGCTG (Fwd)
	CAGCGCGACCCGGTCGCGGC**GGC**GTACTCGCCGGAGCGGACGC (Rev)
G137A	GCGAGTACGACGCCGCGACC**GCC**TCGCGCTGGATGCCCGACCGC (Fwd)
	GCGGTCGGGCATCCAGCGCGA**GGC**GGTCGCGGCGTCGTACTCGC (Rev)
S138A	GAGTACGACGCCGCGACCGGG**GCC**CGCTGGATGCCCGACCGCGTC (Fwd)
	GACGCGGTCGGGCATCCAGCG**GGC**CCCGGTCGCGGCGTCGTACTC (Rev)
R139A	GAGTACGACGCCGCGACCGGGTCG**GCC**TGGATGCCCGACCGCGTCGCCGAC (Fwd)
	GTCGGCGACGCGGTCGGGCATCCA**GGC**CGACCCGGTCGCGGCGTCGTACTC (Rev)
R139D	GAGTACGACGCCGCGACCGGGTCG**GAC**TGGATGCCCGACCGCGTCGCCGACC (Fwd)
	GGTCGGCGACGCGGTCGGGCATCCA**GTC**CGACCCGGTCGCGGCGTCGTACTC (Rev)
R139E	GAGTACGACGCCGCGACCGGGTCG**GAG**TGGATGCCCGACCGCGTCGCCGACC (Fwd)
	GGTCGGCGACGCGGTCGGGCATCCA**CTC**CGACCCGGTCGCGGCGTCGTACTC (Rev)
R139K	GAGTACGACGCCGCGACCGGGTCG**AAG**TGGATGCCCGACCGCGTCGCCGACC (Fwd)
	GGTCGGCGACGCGGTCGGGCATCCA**CTT**CGACCCGGTCGCGGCGTCGTACTC (Rev)
R139M	GAGTACGACGCCGCGACCGGGTCG**ATG**TGGATGCCCGACCGCGTCGCCGACC (Fwd)
	GGTCGGCGACGCGGTCGGGCATCCA**CAT**CGACCCGGTCGCGGCGTCGTACTC (Rev)
R152A	GTCGCCGACCGTCCGCGGAAA**GCC**GGCGTGCCGAGTCGCGCATAC (Fwd)
	GTATGCGCGACTCGGCACGCC**GGC**TTTCCGCGGACGGTCGGCGAC (Rev)
R152D	GTCGCCGACCGTCCGCGGAAA**GAC**GGCGTGCCGAGTCGCGCATAC (Fwd)
	GTATGCGCGACTCGGCACGCC**GTC**TTTCCGCGGACGGTCGGCGAC (Rev)
R152K	GTCGCCGACCGTCCGCGGAAA**AAG**GGCGTGCCGAGTCGCGCATAC (Fwd)
	GTATGCGCGACTCGGCACGCC**CTT**TTTCCGCGGACGGTCGGCGAC (Rev)

D173A	CTCCGCTCGGACCTCCGC**GCC**CACCAGTGCGGCTTCAAG (Fwd)<br>CTTGAAGCCGCACTGGTG**GGC**GCGGAGGTCCGAGCGGAG (Rev)
D173E	CTCCGCTCGGACCTCCGC**GAG**CACCAGTGCGGCTTCAAGG (Fwd)<br>CCTTGAAGCCGCACTGGTG**CTC**GCGGAGGTCCGAGCGGAG (Rev)
D173K	CTCCGCTCGGACCTCCGC**AAG**CACCAGTGCGGCTTCAAGG (Fwd)<br>CCTTGAAGCCGCACTGGTG**CTT**GCGGAGGTCCGAGCGGAG (Rev)
D173N	CTCCGCTCGGACCTCCGC**AAC**CACCAGTGCGGCTTCAAGG (Fwd)<br>CCTTGAAGCCGCACTGGTG**GTT**GCGGAGGTCCGAGCGGAG (Rev)
Q175A	GCTCGGACCTCCGCGACCAC**GCG**TGCGGCTTCAAGGCGTTCAG (Fwd)<br>CTGAACGCCTTGAAGCCGCA**CGC**GTGGTCGCGGAGGTCCGAGC (Rev)
C176A	CGGACCTCCGCGACCACCAG**GCC**GGCTTCAAGGCGTTCAGCCGCG (Fwd)<br>CGCGGCTGAACGCCTTGAAGCC**GGC**CTGGTGGTCGCGGAGGTCCG (Rev)
G177A	GACCTCCGCGACCACCAGTGC**GCC**TTCAAGGCGTTCAGCCGCGAG (Fwd)<br>CTCGCGGCTGAACGCCTTGAA**GGC**GCACTGGTGGTCGCGGAGGTC (Rev)
F178A	GACCTCCGCGACCACCAGTGCGGC**GCC**AAGGCGTTCAGCCGCGAGGCG (Fwd)<br>CGCCTCGCGGCTGAACGCCTT**GGC**GCCGCACTGGTGGTCGCGGAGGTC (Rev)
K179A	GCGACCACCAGTGCGGCTTC**GCG**GCGTTCAGCCGCGAGGCGTTC (Fwd)<br>GAACGCCTCGCGGCTGAACGC**CGC**GAAGCCGCACTGGTGGTCGC (Rev)
D195A	GCTCCGCGACGACGTAGAG**GAG**AACCACTGGTTTTGGGACAC (Fwd)<br>GTGTCCCAAAACCAGTGGTT**CTC**CTCTACGTCGTCGCGGAGC (Rev)
D195E	GCTCCGCGACGACGTAGAG**GAG**AACCACTGGTTTTGGGACAC (Fwd)<br>GTGTCCCAAAACCAGTGGTT**CTC**CTCTACGTCGTCGCGGAGC (Rev)
D201A	GAGGACAACCACTGGTTTTGG**GCC**ACGGAGATGCTCGTCCGCGCCC (Fwd)<br>GGGCGCGGACGAGCATCTCCGT**GGC**CCAAAACCAGTGGTTGTCCTC (Rev)
D201E	GAGGACAACCACTGGTTTTGG**GAG**ACGGAGATGCTCGTCCGCGCCC (Fwd)<br>GGGCGCGGACGAGCATCTCCGT**CTC**CCAAAACCAGTGGTTGTCCTC (Rev)
D201K	GAGGACAACCACTGGTTTTGG**AAG**ACGGAGATGCTCGTCCGCGCCC (Fwd)<br>GGGCGCGGACGAGCATCTCCGT**CTT**CCAAAACCAGTGGTTGTCCTC (Rev)
D201N	GAGGACAACCACTGGTTTTGG**AAC**ACGGAGATGCTCGTCCGCGCCC (Fwd)<br>GGGCGCGGACGAGCATCTCCGT**GTT**CCAAAACCAGTGGTTGTCCTC (Rev)

Fwd – forward primer; Rev – reverse primer. The codon containing the introduced mutation is shown in bold.
